# Evaluation of Salivary GAPDH as a Predictor Biomarker for Periodontitis

**DOI:** 10.3390/ijms262110441

**Published:** 2025-10-27

**Authors:** Elisa Bellei, Stefania Bergamini, Roberta Salvatori, Carlo Bertoldi

**Affiliations:** 1 Proteomic Laboratory, Department of Surgery, Medicine, Dentistry and Morphological Sciences with Transplant Surgery, Oncology and Regenerative Medicine Relevance, University of Modena and Reggio Emilia, Via G. Campi 287, 41125 Modena, Italy; stefania.bergamini@unimore.it; 2 Cranio-Maxillo-Facial Surgery, Department of Medical and Surgical Sciences for Children and Adults, University of Modena and Reggio Emilia School of Medicine, Via del Pozzo 71, 41124 Modena, Italy; roberta.salvatori@unimore.it; 3 Unit of Dentistry and Oral-Maxillofacial Surgery, Periodontology Section, Department of Surgery, Medicine, Dentistry and Morphological Sciences with Transplant Surgery, Oncology and Regenerative Medicine Relevance, University-Hospital of Modena and Reggio Emilia, Via del Pozzo 71, 41124 Modena, Italy; carlo.bertoldi@unimore.it

**Keywords:** GAPDH, periodontitis, saliva, biomarker, proteomics, electrophoresis, mass spectrometry

## Abstract

Periodontitis (PD) is a multifactorial, progressive inflammatory disease affecting the teeth-supporting tissues, characterized by an imbalance of the oral microbiota and the presence of bacterial biofilms leading to host response. Nowadays, reliable biochemical markers for early and objective diagnosis, and for predicting disease progression, are still lacking. Our previous proteomic investigations revealed the significant overexpression of glyceraldehyde-3-phosphate dehydrogenase (GAPDH) in periodontal pocket tissue, gingival crevicular fluid (GCF), and tooth-surface-collected material (TSCM) from PD patients in comparison to periodontally healthy controls, proposing it as a possible biomarker of PD. This study aimed to evaluate the expression of GAPDH in saliva, a more accessible, non-invasive, and clinically relevant oral sample. The whole saliva was analyzed by a preliminary mass spectrometry-based proteomic approach, identifying significantly increased levels of GAPDH also in salivary samples from periodontal-affected subjects. These data were further validated by enzyme-linked-immunosorbent assay (ELISA). Additionally, protein–protein interaction networks were generated through the Human Protein Atlas database, using different datasets (OpenCell, IntAct, and BioGRID). Bioinformatic analysis provided noteworthy GAPDH-associated networks potentially relevant to periodontal pathology. The scientific significance of this study lies in the detection of salivary GAPDH as a novel strategy to advance periodontal clinical diagnostics from the perspective of a non-invasive screening test. In correlation with other protein markers, salivary GAPDH could constitute a promising set of distinctive and predictive targets to enhance early diagnosis of PD, disease monitoring, and treatment planning in periodontology.

## 1. Introduction

The first findings relating to the glyceraldehyde-3-phosphate dehydrogenase (GAPDH) enzyme date back to the first half of the last century [1]. In normal cellular conditions, the human cytoplasmic form of GAPDH exists principally as a tetramer of four equal 37 kDa subunits, arranged in a quaternary assembly [2,3]. The homotetramer is also defined as a dimer of dimers, with three 2-fold-symmetry axes [4,5,6]. The crystal structure was determined by X-ray diffraction, and high-resolution ribbon drawing of the GAPDH tetramer highlights the tridimensional configuration of the four subunits, each of which comprises two conserved functional domains [3]. Specifically, human GAPDH contains 335 amino acids and constitutes an N-terminal domain, comprising the NAD^+^-binding site (residues 1–150 and 317–335), and a highly conserved catalytic domain, containing the substrate-binding site (amino acids 151–316), corresponding to the C-terminal domain [5]. GAPDH is ubiquitously expressed in nature, is abundant in cells, and is found in all domains of life. For a long time, it was considered just a housekeeping glycolytic enzyme, mainly present in the cytoplasm, with a key role in glycolysis and gluconeogenesis [1]. Effectively, GAPDH is a highly conserved, NAD^+^-dependent, catalytic enzyme within the glycolytic pathway. It reversibly catalyzes an essential energy-yielding reaction in carbohydrate metabolism, that is, the 6th step of the aerobic glycolytic pathway, namely the reversible oxidative phosphorylation of D-glyceraldehyde-3-phosphate to 1,3-diphospho-glycerate, in the presence of NAD+ (as co-factor, in oxidized form) and inorganic phosphate (as co-substrate), with the production of NADH + 2H+ (NAD in reduced form and two hydrogen ions, respectively) (InterPro database). The discovery of GAPDH in other cellular compartments besides the cytoplasm, including the nucleus, plasma membrane, and mitochondria, has motivated an in-depth exploration of its function and meaning in both human health and disease conditions [7]. It has been defined as a multifaceted protein, or a “moonlighting protein” [8,9], since, in addition to its primary well-outlined role in glycolysis, it was found involved also in several non-metabolic processes, depending on its subcellular localization, such as apoptosis [10,11], intracellular signaling and transduction, abiotic stress [7,12], endocytosis [13], control of gene expression [14], DNA replication and repair [15], modulation of mRNA stability [16], regulation of energy metabolism [17], oxidative stress response [18], and membrane trafficking [19]. These activities are essentially regulated by biomolecular interactions, protein oligomerization, changes in cellular location, and posttranslational modifications, such as phosphorylation, nitrosylation, acetylation, and *N*-acetylglucosamine modification [20]. Other studies also provide evidence that GAPDH is implicated in various diseases, including cancer [21,22,23,24], neurodegenerative disorders [25,26,27,28], and those of pathogenic origin [4]. Therefore, human GAPDH is directly considered a useful molecular target for the treatment of numerous diseases [29].

Periodontitis (PD) is a chronic inflammation and a serious infection that affects and damages the tooth-supporting components, such as the periodontal ligament, alveolar bone, and gingival tissue around teeth [30]. In severe and aggressive forms, this condition can be worsened by the formation of a deep periodontal pocket, leading to tooth loss. Usually, the development of periodontal disease begins with the formation of bacterial plaque by microbial pathogens, and, if left untreated, can evolve over time into PD [31]. Despite advances in technology and growing awareness of good oral hygiene practices, PD still represents a public health problem today. Particularly severe PD is estimated as the sixth most prevalent disease worldwide [32]. Unfortunately, although progress has been made in various research areas, such as microbiology and genetics, to date, the periodontal diagnosis is still based on clinical examination and radiological imaging; a hopeful future perspective is molecular diagnostics [33]. From this point of view, the highly interdisciplinary research field of oral proteomics can be applied in dentistry [34] to deepen the knowledge of PD, as it enables the identification of proteins in cells, tissues, and body fluids, allowing us to gain a comprehensive understanding of the molecular mechanisms underlying pathologies and to discover novel biomarkers [35,36]. In the proteomic era, several oral samples were analyzed in an attempt to characterize oral biomarkers for PD, such as periodontal gingival tissue and gingival crevicular fluid (GCF), tooth pulp, dentin, root canal content, secretion from salivary glands, periodontal ligament, dental stem cells, and saliva [37]. Undoubtedly, the latter is the most advantageous and attractive sample among those mentioned above, since it is easily and abundantly accessible, and its collection is totally safe and non-invasive for patients, with a good cost–benefit ratio [38]. Moreover, whole saliva contains proteins from salivary glands, epithelial cells, and microorganisms, thus providing an overall assessment of periodontal status. In addition, it contains most of the serum constituents, so it can provide valuable information, not only regarding the oral cavity status (for example, dental caries, periodontal diseases, oral cancer) but also about the systemic disease status [39]. However, the proteomic analysis of human saliva is challenging due to the wide dynamic range of protein abundances, spanning several orders of magnitude [40]. To overcome this drawback, different depletion and fractionation techniques have been developed to maximize the coverage of the salivary proteome, enriching the detection of the low-abundance fraction. Nevertheless, depletion methods may lead to the concomitant removal of non-targeted molecules, as previously demonstrated [41].

Currently, salivary diagnostics is applied in numerous conditions, such as autoimmune diseases (Sjogren’s syndrome, multiple sclerosis, sarcoidosis), genetic disorders (e.g., cystic fibrosis), bacterial, viral, and fungal infections, cardiovascular diseases, malignancy, metabolic bone disorder, and renal diseases, as well as for drug-level monitoring, occupational and environmental medicine, psychological stress research, and forensic evidence [42,43]. Recently, inflammatory mediators present in the oral fluids and blood samples of type 1 diabetes mellitus adult patients diagnosed with periodontal disease have been proposed as novel diabetes biomarkers, and as indicators of the patient’s degree of susceptibility to worsening of the periodontal state [44].

In our previous proteomic works conducted on different oral samples obtained from patients with PD, namely, periodontal pocket gingival tissue [37,45], GCF [46], and tooth-surface-collected material (TSCM) [47], we reported the overexpression of a common protein, that is, GAPDH. 

The aim of the present preliminary study was to search for GAPDH in saliva samples from periodontopathic subjects compared to periodontally healthy controls, to assess the presence of this molecule in a more advantageous oral sample. The analyses were performed by electrophoresis (SDS-PAGE) coupled to liquid chromatography–mass spectrometry (LC-MS/MS), and through enzyme-linked immunosorbent assay (ELISA test) as an orthogonal validation and quantification method. Additionally, GAPDH-associated protein interaction networks were searched and created from the Human Protein Atlas (HPA) database.

## 2. Results

### 2.1. Protein Band Analysis and GAPDH Identification by LC-MS/MS

After salivary proteins’ separation by SDS-PAGE, the densitometric analysis of the protein bands by the QuantityOne 1D image analysis software revealed a significantly different band at 37 kDa between healthy and pathological samples (the original, uncropped, and unprocessed gel image is reported in Supplementary Figure S1). Specifically, the band intensity level (expressed as OD × volume) was found to be significantly higher in periodontal patients (0.149 ± 0.036, mean ± SD) in comparison to healthy control subjects (0.095 ± 0.023, mean ± SD), resulting in a *p*-value of 0.018. 

The protein mixture extracted from the 37 kDa band was subjected to LC-MS/MS analysis, thus leading to the identification of GAPDH in the salivary proteome of PD patients and controls. The identification was achieved by LC-ESI-QO-MS/MS mass spectrometer, through the analysis of raw data by a Mascot MS/MS ion search, with the SwissProt database. GAPDH results were identified with the entry name G3P_HUMAN, P04406 as the primary accession number, and with an experimental monoisotopic mass (M_r_) of 36201 Da. The exponentially modified protein abundance index (emPAI), which estimates the absolute content of a given protein in a complex mixture, was 94.87 in healthy controls and 140.76 in periodontopathic patients, respectively, with a 1.5-fold increase (Table 1). The number of significant matching peptides, as well as the number of significant sequences, are reported in the following columns.

**Figure 1:**
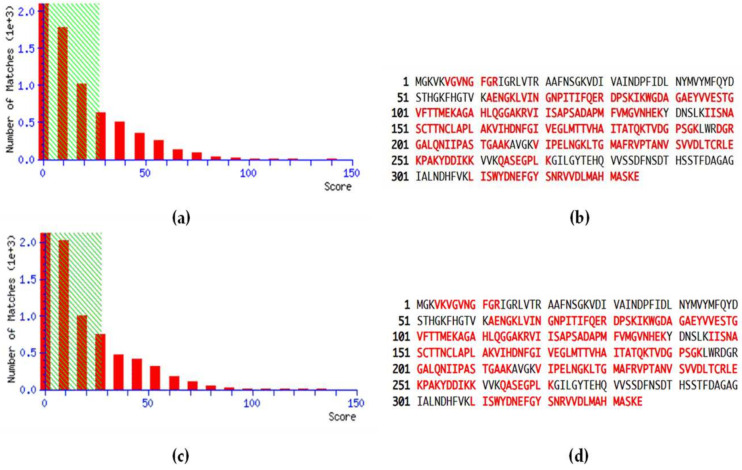
Peptide score distribution resulting from the search in the SwissProt database for GAPDH in healthy samples (**a**) and periodontopathic samples (**c**). The *x*-axis indicates the probability-based mowse score, and the *y*-axis the numbers of hits. Protein sequence coverage: the matching peptides are shown in bold red, corresponding to 68% in healthy controls (**b**) and 67% in PD patients (**d**).

MS data related to the peptide score distributions are shown in Figure 1, for healthy (Figure 1a) and pathological samples (Figure 1c). The peptide score distribution is represented by the ion score, where ion score is −10log(P), and P is the probability that the observed match is a random event. On average, individual ion scores greater than 27 (beyond green shading) indicate identity or extensive homology (*p* < 0.05). Moreover, a high percentage of peptide sequence coverage was achieved for both healthy controls (Figure 1b) and pathological patients (Figure 1d), corresponding to 68% and 67%, respectively.

Some representative MS/MS fragmentation spectra of matching prototypic peptides, used for the identification of GAPDH, are reported in Supplementary Figure S2.

### 2.2. Salivary GAPDH Quantification and Validation 

**Figure 2:**
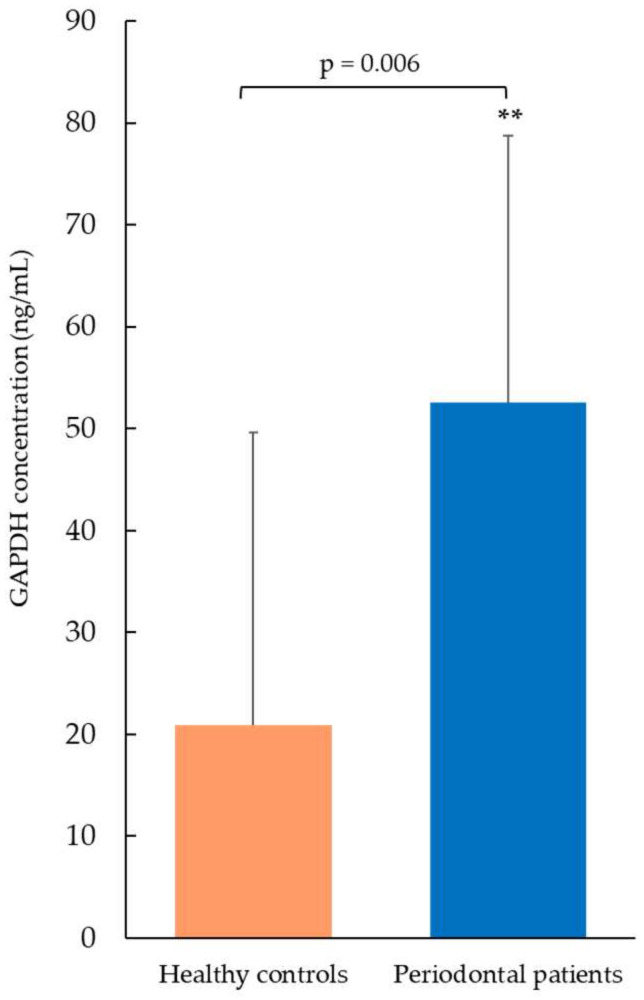
GAPDH concentration measured by ELISA test. Periodontal patients showed significantly increased salivary GAPDH levels compared to healthy controls (** *p* < 0.01). Data are expressed as mean ± SD, and statistical differences were obtained by Student’s *t*-test.

The GAPDH concentration was measured in saliva samples by ELISA (Figure 2). Periodontal patients showed significantly higher levels of GAPDH (52.6 ng/mL ± 26.1, mean ± SD) compared to the healthy control group (20.9 ng/mL ± 28.7, mean ± SD, *p*-value = 0.006). These findings were consistent with those obtained by proteomic analysis. A 7-point standard calibration curve, ranging from 1.56 to 100 ng/mL, was applied to calculate sample concentrations. All salivary values, both from healthy controls and pathological patients, fell within the limits of the calibration curve, and therefore no sample needed to be diluted and re-tested. 

### 2.3. GAPDH Identification in Our Previous Proteomic Studies

GAPDH was previously identified in our proteomic studies performed in different oral samples (Table 2). It was identified in periodontal pocket gingival tissue by two-dimensional gel electrophoresis (2-DE) coupled to Nano LC-ESI-Q-ToF, using the UniProt database [45], and confirmed through SDS-PAGE in association with LC-ESI-QO-MS/MS analysis, using the neXtProt database for identification results [37]. Moreover, GAPDH was detected in GCF samples of patients with severe PD, with a +2.00-fold change in expression in diseased sites compared to periodontally healthy ones [46]. Additionally, LC-MS/MS analysis of both protein bands and spots, obtained from TSCM samples of patients diagnosed with PD and candidates for resective bone therapy, revealed different isoforms of GAPDH [47]. 

### 2.4. Interaction Networks 

**Figure 3:**
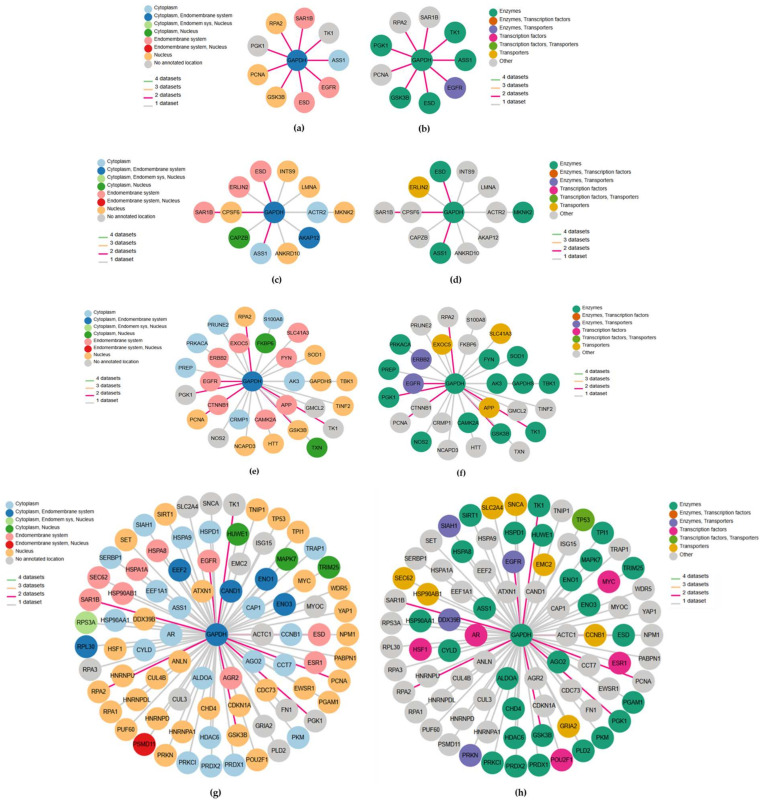
GAPDH interaction networks from different databases colored according to subcellular location and protein class, respectively, for the first-level consensus (**a**,**b**), OpenCell (**c**,**d**), IntAct (**e**,**f**) and BioGRID (**g**,**h**). In each network plot, the nodes correspond to genes and edge color refers to the number of datasets to which the interaction belongs.

The network plots showing the GAPDH-related genes, achieved via the HPA database according to three different datasets, are displayed in Figure 3. 

A first-level consensus network chart was also included. In each network plot, the nodes represent genes and are colored according to their subcellular location (Figure 3a,c,e,g) and protein class (Figure 3b,d,f,h), respectively, while edge colors refer to the number of datasets for the interacting pair. The first-level consensus network showed nine interactions (ASS1, EGFR, ESD, GSK3B, PCNA, PGK1, RPA2, SAR1B, TK1) present in at least two of the queried datasets (Figure 3a,b). With OpenCell, 12 significant GAPDH interaction partners were found (Figure 3c,d), while the molecular interaction database IntAct recognized 29 direct correlations and physical associations with high and medium confidence (Figure 3e,f). Finally, a total of 83 physical multi-validated connections were provided by BioGRID (Figure 3g,h). All the identified GAPDH-interacting key-player genes derived from each dataset, comprising the first-level consensus, are shown in Table 3. The first column refers to the gene symbols (corresponding to those reported in Figure 3), here listed in alphabetical order, followed by the full gene names. The last column shows the main function provided by the HPA database according to gene expression across single cell types or tissue expression cluster. Overall, considering the three queried datasets, a total of 124 key genes interacting with GAPDH were identified (repeated ones are evidenced in italics and are compliant with those of the first-level consensus). Genes previously identified in our PD studies, or those potentially relevant to periodontal disease, are highlighted in bold.

**Figure 4:**
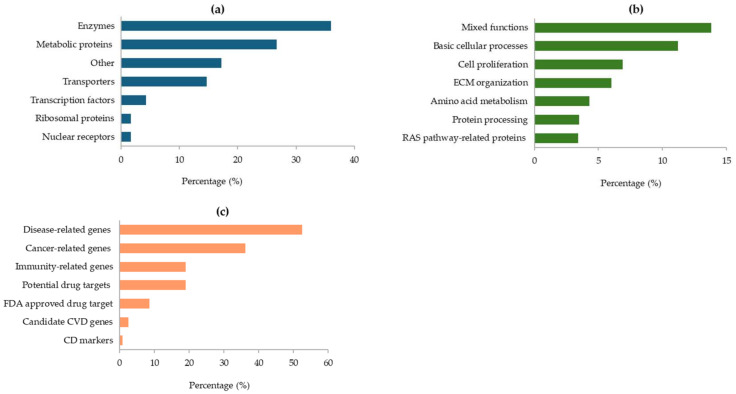
Protein classes assigned by the HPA database for proteins encoded by GAPDH-related genes (**a**); main cellular functions of the identified proteins (**b**); roles of GAPDH-related genes in human diseases (**c**). All data are derived from the HPA database.

As illustrated in Figure 4, most of the proteins encoded by the GAPDH-related genes are enzymes (36%), other metabolic proteins (27%), or transporters and transcription factors (Figure 4a). At cellular level, their main functions are essentially linked to basic cellular processes, cell proliferation, and organization of the extracellular matrix (ECM) in the connective tissue. Furthermore, some proteins are involved in amino acids metabolism and protein processing, while others are related to the RAS pathway (Figure 4b). In addition, many of the GAPDH-associated genes were found to be correlated with various diseases (53%) and the immune response (19%). Notably, several genes are potential drug targets, and some of them have already been approved by the FDA (Figure 4c).

Interestingly, proteins involved in immunity, ECM organization, amino acid metabolism, and protein processing were previously reported in our proteomic studies on PD [37], as further discussed below.

## 3. Discussion

Recent advances in mass spectrometry-based proteomics methodologies have opened new avenues to acquiring relevant information in the dental field [34]. In this regard, saliva is the chief oral biofluid in terms of importance and interest in clinical diagnostics, and *Salivaomics* is emerging as a new tool able to offer substantial advantages to deepen the study of oral diseases [48,49]. Over the past few years, salivary proteomic research applied to PD has encompassed different applications, including the understanding of the disease mechanisms, as well as the discovery of periodontal-specific biomarkers [39,50]. Currently, the use of salivary biomarkers in routine clinical settings is still evolving, and there is growing interest in their potential, mainly due to the non-invasive nature of saliva collection, ease of repeating sampling, and relatively low cost [51]. 

The purpose of the present proteomic work was to investigate GAPDH as a possible PD-related biomarker. Detection, quantification, and validation data were obtained by SDS-PAGE (Supplementary Figure S1) and LC-MS/MS analysis (Figure 1, Table 1) and through the ELISA test (Figure 2), respectively, demonstrating a significant overexpression of salivary GAPDH in PD patients compared to periodontally healthy control subjects. In addition to its primary activity in glycolysis, the eukaryotic form of GAPDH has multiple known functions. For example, it has been demonstrated that its accumulation within the nucleus induces cellular apoptosis, as it takes part in the gene expression program and transcriptional activation [7,11,52]. The finding of high GAPDH levels in oral samples from patients with PD may be associated with the destruction of the periodontal gingival tissue caused by intensified cell death. In support of this hypothesis, increased apoptosis events and apoptotic pathways were previously observed by other authors in periodontal gingival tissue, even if the exact mechanisms related to these processes remain unclear [53,54]. The occurrence of apoptotic cells, principally neutrophils, in the gingival tissue of adult subjects with chronic PD in comparison with healthy donors, was demonstrated by Gamonal et al., suggesting that apoptosis is implicated in the pathogenesis of the disease, and that apoptotic processes are involved in the inflammatory response associated with periodontal tissue destruction [55]. The presence of GAPDH could also be combined with the synthesis of pro-apoptotic proteins, like BAX, c-JUN, and GAPDH itself (InterPro database), inducing danger signals and the consequent stimulation of proinflammatory processes, typical of PD. Moreover, GAPDH aggregation mediates cell death under oxidative stress [56]. It has been proven that GAPDH is a key mediator of many oxidative stress responses, involving its nuclear translocation and induction of apoptosis [12]. Cytosolic GAPDH may work as a sensor for redox signals and an information center, with the aim of transducing these signals into appropriate reactions [57]. GAPDH is also involved in other nuclear events, such as DNA replication or repair, RNA export/transport, membrane fusion, and microtubule clustering (HPA database). In this regard, GAPDH binding to actin and tropomyosin may have a role in cytoskeleton assembly (InterPro database). Elevated amounts of actin [45,58] and GAPDH [45] were previously detected in our proteomic studies conducted in periodontal pocket tissue, probably indicating an attempt to restore the damaged gingival tissue. An actin-related protein was also found in the present study by the HPA database (Figure 3, Table 3).

Several oral bacteria exhibit GAPDH on their surface, where it acts as an adhesin. Surface GAPDH binds host ECM proteins (e.g., fibronectin, laminin) and plasminogen, which can promote initial colonization and tissue invasion. Oral streptococci (early colonizers) use GAPDH in co-aggregation and matrix binding, which may facilitate later colonizers associated with PD [59]. Long fimbriae of *Porphyromonas gingivalis* (*P. gingivalis*) bind human GAPDH and interact with streptococcal surface GAPDH (e.g., *S. oralis*), promoting the formation of mixed biofilms and epithelial invasion, key early events in periodontal pathogenesis [60]. Moreover, bacterial surface moonlighters (including GAPDH in multiple species) act as plasminogen receptors; once converted to plasmin, this promotes fibrin/ECM degradation and pathogen tissue penetration, mechanisms also observed in organisms relevant to periodontal ecology [61]. In addition, GAPDH plays a role in microbial virulence, as it is involved in evading host immune surveillance. Because of this property, GAPDH has been suggested as an eligible target for a vaccine to limit diseases caused by various pathogens [62]. This assumption could be applied to PD as well, since it is triggered by bacterial microorganisms, or groups of specific microorganisms. On the other hand, GAPDH also exhibits antimicrobial functions during epithelial infection, protecting tissue through antifungal strategies and immunomodulation [63]. Furthermore, it participates in innate immunity, by promoting TNF-induced NF-kB activation and type I interferon production [64]. In accordance with these activities, the up-regulation of salivary GAPDH detected in PD-affected patients might enhance immune stimulation for defensive and/or reparative purposes against the ongoing disease, thus becoming a new potential therapeutic factor. An active involvement of the immune system was also confirmed by the interaction networks, since a considerable number of GAPDH-associated genes were linked to the innate or adaptive immune response (Table 3). Based on these assumptions, the high amount of salivary GAPDH found in PD could originate from host cell secretion or cell dead release (e.g., neutrophils, gingival cells), active secretion, or bacterial production (e.g., *S. oralis, P. gingivalis*).

Additionally, the implication of GAPDH in different diseases has been widely documented, comprising those of cardiovascular, diabetic, degenerative, and pathogenic origins [4]. Accordingly, GAPDH may have a possible role in PD due to the microbial pathogenic origin of this disease. The involvement of GAPDH has also been proved in some neurodegenerative disorders, such as Alzheimer’s, Parkinson’s, Machado–Joseph, and Huntington’s diseases, in association with abnormal neuronal apoptosis. GAPDH can bind the gene products of these diseases through the interaction with RNA and other proteins, thus improving neuronal survival by reducing the synthesis of pro-apoptotic proteins (InterPro database). From this perspective, GAPDH could also be considered a potential therapeutic target in PD.

In this work, the interaction networks between GAPDH and other proteins/genes were widely investigated. Protein–protein interactions are crucial for several cellular mechanisms, such as gene expression and control, antibody–antigen binding, and cell signaling. Frequently, the alteration or blockage of these ways can lead to diseases, so knowledge of the interaction networks can contribute to the understanding of pathological processes. The HPA is the largest and most comprehensive database for protein spatial distribution in cells and tissues, generating spatial maps of human protein-coding genes [65]. The interaction networks for GAPDH were based on data from OpenCell, IntAct, and BioGRID datasets, integrated with data related to protein function and classification (Figure 3, Table 3). Notably, some proteins encoded by these genes were previously detected in our proteomic studies conducted on various oral samples from PD patients. Peroxiredoxin 1 (PRDX1), peroxiredoxin 2 (PRDX2), and triosephosphate isomerase (TPI1), together with some related protein forms (like protein S100-A9, alpha enolase, ENOA, and the beta-1 form of heat shock protein, HSPB1) were found up-regulated in the first preliminary proteomic analysis performed directly on periodontal pocket tissue [45]. Then, the eukaryotic translation elongation factor 1 alpha 1 (EEF1A1), the enzyme pyruvate kinase (PKM2), as well as other protein partners, such as Hsp60 and Hsp70, were detected in TSCM proteome of periodontopathic patients by 2-DE coupled to LC-MS/MS analysis [47]. In addition, EEF1A1 was also identified in pocket-associated gingival tissue from patients with severe PD, concurrently with fibronectin 1 (FN1) [37]. Recently, in our latest proteomic work carried out on saliva samples, PRDX1, TPI1, thioredoxin (TXN), phosphoglycerate mutase 1 (PGAM1), and correlated forms of superoxide dismutase (SOD2), peroxiredoxin-6 (PRDX6), protein S100-A9, heat shock proteins (HSPB1, Hsp90-beta), and proteasome (subunits alpha and beta), were revealed to be overexpressed in patients with advanced PD when compared to periodontally healthy subjects [66]. Therefore, the present study contributed to further supporting their biological and diagnostic relevance, validating the arguments in favor of using these key players as focal targets for the treatment of PD. Likewise, the crucial mediators interacting with GAPDH mainly included enzymes, as also reported in saliva [66], proteins involved in ECM organization in connective tissue, as found in damaged periodontal pocket gingival tissue [37], and several immunity-related proteins (Figure 4). Various forms of immunoglobulins were found early on, especially in GCF [46] and periodontal tissue [37]. It is remarkable to note that certain GAPDH-related genes reported in Table 3 are recognized as potential drug targets (e.g., ERLIN2, implicated in salivary secretion, SOD1, ENO3, HSPD1, TPI1), and some of these are already approved by the FDA (e.g., EEF2 and FN1) (data inferred from the HPA database). 

A comprehensive understanding of the molecular determinants and signaling pathways involving GAPDH might allow its potential rational targeting as a modulation/regulation point or selective biomarker for predictive and therapeutic applications in pathological conditions, such as PD. Advances in proteomic-based technologies promise to constantly help in understanding what is still unexplored in oral health and disease, gradually leading to the transition from laboratory research to clinical practice, through the discovery and validation of potential biochemical markers for use in daily saliva-based testing, in addition to clinical scores [67]. 

At present, PD is diagnosed after periodontal damage has already occurred. Moreover, the diagnosis is subjective and must be performed by trained and experienced operators to be validated. The best periodontal diagnosis of the future should be, above all, an early diagnosis, made before such damage occurs, and should combine radiological, clinical, and laboratory assays [33]. Accordingly, the unbiased protein biomarkers identified in our studies could support the feasibility of translating proteomic findings into practical high-throughput screening tests, such as saliva-based protein microarrays, for an objective early diagnosis and desirable routine periodontal monitoring.

### 3.1. Final Remarks 

GAPDH is more than just a glycolytic enzyme in the periodontal niche. In PD, it may contribute to colonization by *P. gingivalis* and other pathogens, immune evasion, and proinflammatory signaling. This is a classic example of a bacterial housekeeping enzyme, which acquires moonlighting roles in pathogenesis, helping bacteria survive in the hostile periodontal pocket while amplifying the destruction of host tissue. 

GAPDH was found to be overexpressed in all types of oral samples previously analyzed (Table 2), namely, periodontal pocket gingival tissue [37,45], GCF [46], and TSCM [47], and now that is also confirmed in saliva of patients with severe PD. Furthermore, several important GAPDH-interacting proteins, such as PRDX1, PRDX2, TPI1, EEF1A1, FN1, PKM2, TXN, and PGAM1, earlier detected in other proteomic studies, were confirmed by data from the HPA database analysis, together with some partner proteins (i.e., ENO1, ENO3, protein S100-A8, SOD1, HSPD1, other forms of Hsp60, Hsp70, and proteasome). These are key findings, as the use of multiple biomarkers, representing different components and mechanisms of complex disease pathways, can provide a broader evaluation of the disease itself [68]. A good biomarker is not conceived as a single gold standard, but rather as a cluster of multiple molecules. 

### 3.2. Limitations and Future Directions

Further validations are needed to assess disease specificity, comparing the expression profiles in other oral inflammatory conditions, such as gingivitis or mucositis in longitudinal studies, to better define the discriminatory potential of these candidate biomarkers. Subsequent confirmation of the current preliminary results in a larger, independent cohort will be planned. This could allow for the calculation of diagnostic performance metrics, such as sensitivity, specificity, and ROC curve/AUC, to perform a diagnostic efficacy evaluation. Moreover, additional key objectives will be to define the source of elevated salivary GAPDH concentrations found in PD, as well as to experimentally validate the specific mechanisms in which it is involved.

## 4. Materials and Methods

### 4.1. Materials 

Dithiothreitol (DTT), iodoacetamide, 2X Laemmli sample buffer, ampholyte pH 3–10, Protein assay Dye Reagent, Precision Plus Protein Standard - All Blue, and Coomassie Blue G-250 were purchased from Bio-Rad Laboratories (Milan, Italy). Trichloroacetic acid, urea, thiourea, trifluoroacetic acid, 3-[(3-Cholamidopropyl)-dimethylammonium]-propane-sulfonate (CHAPS), bovine serum albumin, and 2-mercaptoethanol were from Merck KGaA (Milan, Italy). Precast gel Bolt™ 4–12% Bis-Tris Plus and MES SDS-PAGE Bolt^TM^ Running Buffer were from Life Technologies Italia (Monza, Italy). Trypsin Gold Mass Spectrometry grade was furnished from Promega Corporation (Milan, Italy). The solvents, acetone, acetonitrile, and glacial acetic acid, all of mass spectrometry purity, were from Carlo Erba Reagents (Milan, Italy). Versylene sterile water (Fresenius Kabi, Isola della Scala, Italy) was used for all dilutions, electrophoresis, and MS protocols. 

### 4.2. Enrollment of Study Participants 

The participants in this study were enrolled at the Periodontology Unit of Dentistry and Oral-Maxillofacial Surgery of the University Hospital of Modena, Italy, after approval of the local Health Service Ethic Committee, Emilia-Romagna region (protocol code n. 3968/2017, registration C.E. n. 315/17, date of approval 24 October 2017, and supplementary amendment protocol AOU n. 0026796/22, date of approval 21 September 2022). All the enrolled individuals, selected from January 2023 to February 2024, were fully informed about the procedures, before signing an informed consent to participate, according to the guidelines of the Declaration of Helsinki. 

A total of 30 subjects in good general health were recruited, divided into a control group composed of 20 periodontally healthy individuals (9 males/11 females), with a mean age of 24.7 ± 4.0 years (range 18–32), found free of gingivitis and PD after a dental examination, and a pathological group, consisting of 10 patients with grade III/IV PD [69], (4 males/6 females) with a mean age of 52.9 ± 8.3 years (range 43–65 years), referred to dental and periodontal therapy. The stringent screening criteria included systemically healthy subjects aged between 18 and 65 years, who were non-smokers, not pregnant or lactating, and not undergoing pharmacological treatments (comprising anti-inflammatory drugs and antibiotics), without history of alcohol abuse and in the absence of diabetes, bone diseases, inflammatory conditions, and recent infections [70]. All dental–periodontal procedures and clinical measurements were previously reported in detail [37,46,66].

### 4.3. Saliva Collection and Salivary Protein Extraction and Quantification

To reduce variability due to the circadian rhythm and external factors (such as food, beverages, and oral hygiene products), saliva samples were collected in the morning from subjects who had not eaten, drunk, brushed their teeth, or used mouthwash for at least 30 min prior to collection. This protocol aimed to control for variables in salivary composition between individuals, thus ensuring more accurate data comparability.

Whole saliva samples were harvested using oral salivary swabs SalivaBio (Salimetrics, State College, PA, USA), that were centrifuged at 1500× *g* for 15 min at +4 °C. Then, the concentrated samples were aliquoted in sterile Eppendorf tubes and stored at −80 °C until further analysis. 

Subsequently, proteins were extracted from saliva as described by Jessie et al. [71]. In the first step, saliva samples were precipitated overnight at –20 °C, with a precipitation solution composed of 90% acetone/20% trichloroacetic acid (TCA)/20 mM DTT. The protein precipitate, obtained by centrifugation at 15.000× *g*, for 30 min, at +4 °C (Centrisart^®^ A-14C, Sartorius) was first washed with 90% acetone/20 mM DTT, and subsequently with 80% acetone/10 mM DTT. Finally, the protein pellet was resuspended in 30 μL of rehydration buffer, composed of 6 M urea, 2 M thiourea, 4% CHAPS, and 25 mM DTT, 0.2% ampholyte pH range 3–10. 

The total protein content of each sample was quantified by the spectrophotometric Bradford method, using the protein assay dye reagent (Bio-Rad Laboratories, Hercules, CA, USA), and bovine serum albumin was the standard for the calibration curve. Measurements were performed in a microplate reader (Multiskan^TM^ FC, Thermo Fisher Scientific, Waltham, MA, USA), at a wavelength of 595 nm. 

### 4.4. Protein Separation by SDS-PAGE and LC-MS/MS Analysis 

Salivary protein separation was carried out by SDS-PAGE under denaturing conditions. The equivalent of 20 µg of protein from each extract was denatured with 2X Laemmli sample buffer (Bio-Rad), supplemented with 0.5% of 2-mercaptoethanol, by incubation at +95 °C in Thermomixer Comfort (Eppendorf, Milan, Italy). Separation was performed on precast gradient gel Bolt^TM^ 4–12% Bis-Tris Plus (Life Technologies, Monza, MD, Italy) in MES SDS-PAGE Bolt^TM^ Running Buffer (Life Technologies, Monza, MD, Italy) at a voltage of 200 V. The gels were stained overnight with Coomassie Blue G-250, and de-stained with 5% glacial acetic acid. Subsequently, gel images were acquired by a calibrated densitometer (model GS-800, Bio-Rad, Hercules, CA, USA) and then analyzed by QuantityOne 1D image analysis software (version 4.6.7, Bio-Rad, Hercules, CA, USA), which detects protein bands based on their stain intensity. The band density derives from the sum of the intensities of all the pixels that make the band. By eliminating the background noise and correcting for the variability that can result from gel staining, the software allows the detection of differentially expressed protein bands between different samples. 

The differentially expressed bands between periodontal healthy controls and patients with PD were removed from the gel and subjected to the “in-gel digestion” protocol, as previously reported [41]. First, bands were de-stained using acetonitrile (ACN), and then proteins were reduced and alkylated with DTT at +56 °C and with 55 mM iodoacetamide, respectively. Digestion was obtained with Trypsin Gold Mass Spectrometry grade (Promega Corporation, Milan, Italy). Finally, peptides were extracted using trifluoroacetic acid and ACN. Mass spectrometry analysis was performed using a Nano UHPLC-MS Exploris 480 (Thermo Fisher Scientific, Reinach, Switzerland), consisting of a nano UHPLC Ultimate 3000 System coupled with an Exploris™ 480 Hybrid Quadrupole-Orbitrap™ Mass Spectrometer, as previously described [72]. MS data processing was performed with the Mascot search engine (www.matrixscience.com, accessed on 15 September 2023), through an MS/MS ion search for protein identification, based on raw MS/MS data from one or more peptides.

### 4.5. Salivary GAPDH Quantification by ELISA 

The salivary level of GAPDH was quantified through the Human Glyceraldehyde-3-Phosphate Dehydrogenase (GAPDH) ELISA Kit (Invitrogen, provided by Life Technologies Italia, Milan, Italy), following the manufacturer’s guidelines. This is a solid-phase sandwich immunoenzymatic assay, designed to detect and quantify the level of human GAPDH in serum, plasma, and other biological fluids, such as saliva. Briefly, after centrifugation at 1.000× *g* for 20 min at +4 °C, 100 μL of saliva samples and standards were deposited into the plate wells containing the pre-coated specific antibody for GAPDH. The plate was then incubated for 90 min in a stove set at +37 °C, to allow antigen binding. Afterwards, 100 μL of biotinylated detection antibody was dispensed into each well and incubated for further 60 min. The solution was then aspirated, and the wells were washed with the supplied wash buffer, before adding the HRP conjugate solution. After 30 min incubation, the wash process was repeated 5 times with 300 μL of 1X wash buffer before adding 90 μL of substrate reagent to each well, for the last 15 min incubation at +37 °C, protecting the plate from light. Finally, the reaction was blocked by adding 50 μL of stop solution, turning the color from blue to yellow. A microplate reader (Multiscan FC, Thermo Scientific, Waltham, MA, USA), preheated before the measurements, was employed to read the absorbances at a wavelength of 450 nm, within 10 min after adding the stop solution. The background (blank) OD was subtracted from all data points, and a standard calibration curve was used to read sample concentrations. 

### 4.6. GAPDH Interaction Networks

The HPA database (www.proteinatlas.org, accessed on 27 November 2024), a useful tool for protein localization and expression in cells and tissues [65], was used to create GAPDH-related interaction networks according to the following datasets: OpenCell, a proteome-scale measurement of human protein localization and interactions, IntAct, a molecular interaction database that allows us to generate direct correlations and physical associations with high and medium confidence, and BioGRID, a database of protein, genetic, and chemical connections. A consensus network, showing only interactions present in more than one of the datasets, was also included. The nodes were colored based on subcellular location (according to the data present in the subcellular section of the database) and protein class. 

### 4.7. Statistics and Data Processing

Data were statistically analyzed by an independent parametric Student’s *t*-test, considering a *p*-value < 0.05 as significant. All reported data are expressed as mean ± standard deviation (SD). Mascot parameters for protein identification from primary sequence databases using MS data were set as follows: type of search, MS/MS ion search; preferred taxonomy, Homo sapiens (Human); proteolytic enzyme, trypsin; fixed modification, carbamidomethylation (Cysteines); variable modification, deamidation (Asparagine, Glutamine) and oxidation (Methionine); mass values, monoisotopic; protein mass, unrestricted; peptide mass tolerance, ± 10 ppm; fragment mass tolerance, ± 0.02 Da; max trypsin missed cleavages, one; min. number of significant unique sequences, two; significant threshold, *p* < 0.05; databases for detection/exclusion of any contaminants, cRAP, and for peptide sequences, SwissProt, respectively (accessed on 14 September 2023).

## 5. Conclusions

In conclusion, oral clinical proteomics has proved to be a promising tool to explore PD-related changes in the proteome composition of oral samples, such as saliva, thus pointing towards the discovery of selective and reliable markers associated with the disease. These results suggest that, in addition to its numerous well-defined functions, GAPDH may also play a role as a predictive biomarker of PD, as well as for the assessment and monitoring of disease progression and periodontal therapy outcomes. In a non-invasive and rapid salivary screening test, GAPDH could be included in a damage-associated molecular pattern, which could find clinical applications to recognize individuals early on who are at risk of active PD, thus improving the clinical management and treatment of periodontal patients.

## Figures and Tables

**Table 1:** GAPDH identification by LC-MS/MS analysis in saliva samples of controls and periodontal patients.

Sample Type	Entry Name ^(a)^	Acc. No.^(b)^	Mass ^(c)^	emPAI ^(d)^	Match. ^(e)^	Seq. ^(f)^	Score ^(g)^	Cov. ^(h)^
Heathy controls	G3P_HUMAN	P04406	36201	94.87	103	23	2774	68%
PD patients	G3P_HUMAN	P04406	36201	140.76	147	24	4023	67%

^(a)^ Primary protein entry name (SwissProt database); ^(b)^ protein accession number (SwissProt database); ^(c)^ experimental monoisotopic mass, expressed in Dalton; ^(d)^ exponentially modified protein abundance index; ^(e)^ significant matching peptides; ^(f)^ significant sequences; ^(g)^ highest scores from Mascot search engine; ^(h)^ percentage of amino acid sequence coverage.

**Table 2:** Previous identification of GAPDH in different periodontal oral samples by proteomics.

Ref. No.	Authors (Year of Publication)	Type of Oral Sample ^(a)^	Protein Separation Method ^(b)^	Type of Mass Spectrometer ^(c)^	Type of Protein Database ^(d)^
[45]	Monari et al. (2015)	Periodontal pocket tissue	2-DE	Nano LC-ESI-Q-ToF	UniProt
[37]	Bellei et al. (2024)	Periodontal pocket tissue	SDS-PAGE	LC-ESI-QO-MS/MS	neXtProt
[46]	Bellei et al. (2022)	GCF	SDS-PAGE	LC-ESI-QO-MS/MS	neXtProt
[47]	Bergamini et al. (2021)	TSCM	SDS-PAGE + 2-DE	LC-ESI-QO-MS/MS	neXtProt + SwissProt

^(a)^ Type of oral sample analyzed, collected from patients with PD; ^(b)^ electrophoretic method used for protein separation, two-dimensional gel electrophoresis (2-DE) or mono-dimensional gel electrophoresis (SDS-PAGE); ^(c)^ type of mass spectrometer employed for protein identification; ^(d)^ types of queried protein databases.

**Table 3:** List of genes correlated to GAPDH based on different interaction databases.

Gene ^(a)^	Full Gene Name ^(b)^	Main Function ^(c)^
First-level consensus		
*ASS1*	Argininosuccinate synthase 1	Amino acid metabolism
*EGFR*	Epidermal growth factor receptor	ECM organization
*ESD*	Esterase D	Mixed function (metabolism)
*GSK3B*	Glycogen synthase kinase 3 beta	Mixed function (transcription)
*PCNA*	Proliferating cell nuclear antigen	Cell proliferation
*PGK1*	Phosphoglycerate kinase 1	Mixed function
*RPA2*	Replication protein A2	Adaptive immune response
*SAR1B*	Secretion associated Ras related GTPase 1B	Protein processing
*TK1*	Thymidine kinase 1	Cell proliferation
OpenCell database		
*ACTR2*	Actin related protein 2	Degranulation
*AKAP12*	A-kinase anchoring protein 12	Vessel development
*ANKRD10*	Ankyrin repeat domain 10	Non-specific (transcription)
*ASS1*	Argininosuccinate synthase 1	Amino acid metabolism
*CAPZB*	Capping actin protein of muscle Z-line subunit beta	Basic cellular processes
*CPSF6*	Cleavage and polyadenylation specific factor 6	Mixed function (transcription)
*ERLIN2*	ER lipid raft associated 2	Salivary secretion
*ESD*	Esterase D	Mixed function (metabolism)
*INTS9*	Integrator complex subunit 9	Non-specific (transcription)
*LMNA*	Lamin A/C	Innate immune response
*MKNK2*	MAPK interacting serine/threonine kinase 2	Cornificatio
*SAR1B*	Secretion associated Ras related GTPase 1B	Protein processing
IntAct database		
*AK3*	Adenylate kinase 3	Basic cellular processes
*APP*	Amyloid beta precursor protein	Mixed function
*CAMK2A*	Calcium/calmodulin dependent protein kinase II α	Neuronal signaling
*CRMP1*	Collapsin response mediator protein 1	Neuronal signaling
*CTNNB1*	Catenin beta 1	Innate immune response
*EGFR*	Epidermal growth factor receptor	ECM organization
*ERBB2*	Erb-b2 receptor tyrosine kinase 2	Vesicular transport
*EXOC5*	Exocyst complex component 5	Non-specific (transcription)
*FKBP6*	FKBP prolyl isomerase family member 6 (inactive)	Non-specific
*FYN*	FYN proto-oncogene, Src family tyrosine kinase	Immune system, transcription
*GAPDHS*	GAPDH, spermatogenic	Spermatogenesis
*GMCL2*	Germ cell-less 2, spermatogenesis associated	Spermatogenesis
*GSK3B*	Glycogen synthase kinase 3 beta	Mixed function (transcription)
*HTT*	Huntingtin	Basic cellular processes
*NCAPD3*	Non-SMC condensin II complex subunit D3	Cell proliferation
*NOS2*	Nitric oxide synthase 2	Unknown function
*PCNA*	Proliferating cell nuclear antigen	Cell proliferation
*PGK1*	Phosphoglycerate kinase 1	Mixed function
*PREP*	Prolyl endopeptidase	Neuronal signaling
*PRKACA*	Protein kinase cAMP-activated catalytic subunit α	Innate immune response
*PRUNE2*	Prune homolog 2 with BCH domain	Endocytosis
*RPA2*	Replication protein A2	Adaptive immune response
*S100A8*	S100 calcium binding protein A8	Cornification
*SLC41A3*	Solute carrier family 41 member 3	Non-specific (transcription)
*SOD1*	Superoxide dismutase 1	Amino acid metabolism
*TBK1*	TANK binding kinase 1	Innate immune response
*TINF2*	TERF1 interacting nuclear factor 2	Adaptive immune response
*TK1*	Thymidine kinase 1	Cell proliferation
*TXN*	Thioredoxin	Innate immune response
BioGRID database		
*ACTC1*	Actin alpha cardiac muscle 1	Muscle contraction
*AGO2*	Argonaute RISC catalytic component 2	Innate immune response
*AGR2*	Anterior gradient 2, protein disulfide isomerase	Mucin production
*ALDOA*	Aldolase, fructose-bisphosphate A	Non-specific (transcription)
*ANLN*	Anillin, actin binding protein	Myelination
*AR*	Androgen receptor	Transcription
*ASS1*	Argininosuccinate synthase 1	Amino acid metabolism
*ATXN1*	Ataxin 1	Unknown function
*CAND1*	Cullin associated and neddylation dissociated 1	Non-specific (transcription)
*CAP1*	Cyclase associated actin cytoskeleton regulatory protein 1	Innate immune response
*CCNB1*	Cyclin B1	Cell proliferation
*CCT7*	Chaperonin containing TCP1 subunit 7	Basic cellular processes
*CDC73*	Cell division cycle 73	Unknown function
*CDKN1A*	Cyclin dependent kinase inhibitor 1A	ECM organization
*CHD4*	Chromodomain helicase DNA binding protein 4	Adaptive immune response
*CUL3*	Cullin 3	Immune system, transcription
*CUL4B*	Cullin 4B	Transcription
*CYLD*	CYLD lysine 63 deubiquitinase	Immune system, transcription
*DDX39B*	DExD-box helicase 39B	Non-specific (transcription)
*EEF1A1*	Eukaryotic translation elongation factor 1 alpha 1	Non-specific
*EEF2*	Eukaryotic translation elongation factor 2	Non-specific
*EGFR*	Epidermal growth factor receptor	ECM organization
*EMC2*	ER membrane protein complex subunit 2	Mitochondrial translation
*ENO1*	Enolase 1	Basic cellular processes
*ENO3*	Enolase 3	Muscle contraction
*ESD*	Esterase D	Mixed function (metabolism)
*ESR1*	Estrogen receptor 1	Transcription factor
*EWSR1*	EWS RNA binding protein 1	Mixed function
*FN1*	Fibronectin 1	Muscle contraction
*GRIA2*	Glutamate ionotropic receptor AMPA type subunit2	Neuronal signaling
*GSK3B*	Glycogen synthase kinase 3 beta	Mixed function
*HDAC6*	Histone deacetylase 6	Transcription
*HNRNPA1*	Heterogeneous nuclear ribonucleoprotein A1	Non-specific (ribosome)
*HNRNPD*	Heterogeneous nuclear ribonucleoprotein D	Basic cellular processes
*HNRNPDL*	Heterogeneous nuclear ribonucleoprotein D like	Basic cellular processes
*HNRNPU*	Heterogeneous nuclear ribonucleoprotein U	Innate immune response
*HSF1*	Heat shock transcription factor 1	Basic cellular processes
*HSP90AA1*	Heat shock protein 90 α family class A member 1	Transcription
*HSP90AB1*	Heat shock protein 90 α family class B member 1	Transcription
*HSPA1A*	Heat shock protein family A (Hsp70) member 1A	Mitochondrial translation
*HSPA8*	Heat shock protein family A (Hsp70) member 8	Transcription
*HSPA9*	Heat shock protein family A (Hsp70) member 9	Transcription
*HSPD1*	Heat shock protein family D (Hsp60) member 1	Transcription
*HUWE1*	WWE domain containing E3 ubiquitin protein ligase 1	Basic cellular processes
*ISG15*	ISG15 ubiquitin like modifier	Innate immune response (salivary gland)
*MAPK7*	Mitogen-activated protein kinase 7	Innate immune response
*MYC*	MYC proto-oncogene, bHLH transcription factor	Basic cellular processes
*MYOC*	Myocilin	ECM organization
*NPM1*	Nucleophosmin 1	Non-specific (ribosome)
*PABPN1*	Poly(A) binding protein nuclear 1	Immunoglobulins and histones
*PCNA*	Proliferating cell nuclear antigen	Cell proliferation
*PGAM1*	Phosphoglycerate mutase 1	Mixed function
*PGK1*	Phosphoglycerate kinase 1	Mixed function
*PKM*	Pyruvate kinase M1/2	Mixed function
*PLD2*	Phospholipase D2	Innate immune response
*POU2F1*	POU class 2 homeobox 1	Transcription
*PRDX1*	Peroxiredoxin 1	Basic cellular processes
*PRDX2*	Peroxiredoxin 2	Oxygen transport
*PRKCI*	Protein kinase C iota	Vesicular transport
*PRKN*	Parkin RBR E3 ubiquitin protein ligase	Muscle contraction
*PSMD11*	Proteasome 26S subunit, non-ATPase 11	Mixed function
*PUF60*	Poly(U) binding splicing factor 60	Mitochondrial translation
*RPA1*	Replication protein A1	Basic cellular processes
*RPA2*	Replication protein A2	Adaptive immune response
*RPA3*	Replication protein A3	Amino acid metabolism
*RPL30*	Ribosomal protein L30	Non-specific (ribosome)
*RPS3A*	Ribosomal protein S3A	Non-specific (ribosome)
*SAR1B*	Secretion associated Ras related GTPase 1B	Protein processing
*SEC62*	SEC62 homolog, preprotein translocation factor	Protein processing
*SERBP1*	SERPINE1 mRNA binding protein 1	Basic cellular processes
*SET*	SET nuclear proto-oncogene	Adaptive immune response
*SIAH1*	Siah E3 ubiquitin protein ligase 1	Mixed functions
*SIRT1*	Sirtuin 1	Transcription
*SLC2A4*	Solute carrier family 2 member 4	Muscle contraction
*SNCA*	Synuclein alpha	Oxygen transport
*TK1*	Thymidine kinase 1	Cell proliferation
*TNIP1*	TNFAIP3 interacting protein 1	Mixed function
*TP53*	Tumor protein p53	Cytokine signaling
*TPI1*	Triosephosphate isomerase 1	Mixed function
*TRAP1*	TNF receptor associated protein 1	Basic cellular processes
*TRIM25*	Tripartite motif containing 25	Innate immune response
*WDR5*	WD repeat domain 5	Transcription
*YAP1*	Yes1 associated transcriptional regulator	ECM organization

^(a)^ Official gene symbol, which is typically a short form of the gene name, according to HGNC (HUGO Gene Nomenclature Committee). Gene symbols correspond to those reported in Figure 3; ^(b)^ full gene name according to HGNC; ^(c)^ main function from HPA database. In italics: interactions present in more than one of the datasets. In bold: protein-associated genes previously identified in our PD studies, or potentially relevant to PD.

## Data Availability

The original contributions presented in this study are included in the article and in the Supplementary Material. Further inquiries can be directed to the corresponding author upon reasonable request.

## Supplementary Materials

The following supporting information can be downloaded at https://www.mdpi.com/article/10.3390/ijms262110441/s1.

## Abbreviations

The following abbreviations are used in this manuscript:

ACN	Acetonitrile
2-DE	Two-dimensional gel electrophoresis
DTT	Dithiothreitol
ECM	Extracellular matrix
EEF1A1	Eukaryotic translation elongation factor 1 alpha 1
ELISA	Enzyme-linked immunosorbent assay
emPAI	Exponentially modified protein abundance index
ENOA	Alpha enolase
FN1	Fibronectin 1
GAPDH	Glyceraldehyde-3-phosphate dehydrogenase
GCF	Gingival crevicular fluid
HPA	Human Protein Atlas database
LC-MS/MS	Liquid chromatography–mass spectrometry
PD	Periodontitis
PGAM1	Phosphoglycerate mutase 1
PKM2	Pyruvate kinase
PRDX1	Peroxiredoxin 1
PRDX2	Peroxiredoxin 2
SOD2	Superoxide dismutase, mitochondrial
TPI1	Triosephosphate isomerase
TSCM	Tooth-surface-collected material
TXN	Thioredoxin
