# Supplementary material for: Evaluation of Salivary GAPDH as a Predictor Biomarker for Periodontitis
Source: Int J Mol Sci. 2025 Oct 27;26(21):10441. doi: 10.3390/ijms262110441 (PMC12609646; doi:10.3390/ijms262110441)
Supplement: Supplementary file 1 [file ijms-26-10441-s001.zip › Supplementary Figure S1.pdf]

Supplemenatry Figure S1. Original SDS-PAGE gel image

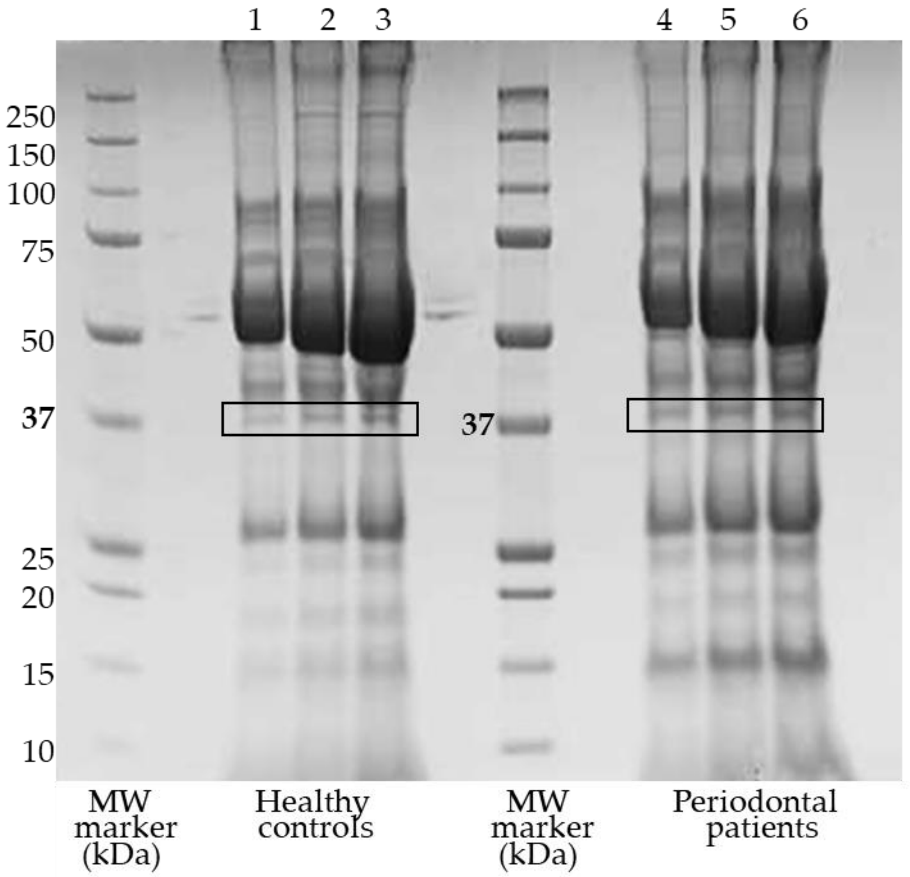

Supplemenatry Figure S1. Original gel image highlighting the band at 37 kDa, resulted differentially expressed between healthy control subjects and periodontal patients, comprising GAPDH as demonstrated by LC-MS/MS analysis. Three pools for healthy controls (lanes 1-3) and three pools for periodontal patients (lanes 4-6) were obtained by combining multiple salivary extracts from the corresponding group. Molecular weight marker expressed in kilodalton (Precision Plus protein standard, all blue, Bio-Rad).
