# Supplementary material for: Evaluation of Salivary GAPDH as a Predictor Biomarker for Periodontitis
Source: Int J Mol Sci. 2025 Oct 27;26(21):10441. doi: 10.3390/ijms262110441 (PMC12609646; doi:10.3390/ijms262110441)

Supplementary Figure S2: Representative MS/MS fragmentation spectra of some prototypical peptides for GAPDH identification in periodontal and healthy salivary samples.

Pathological sample

Healthy sample

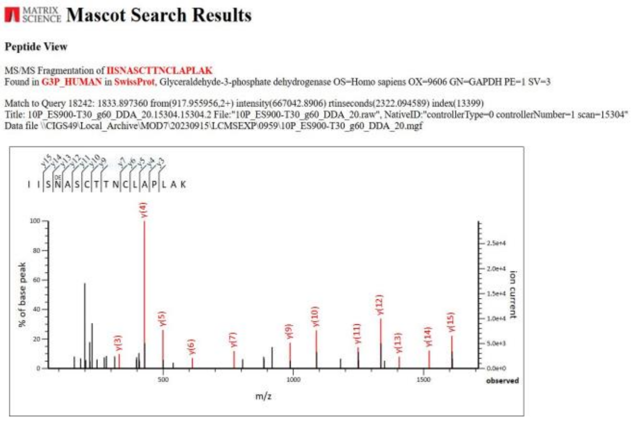

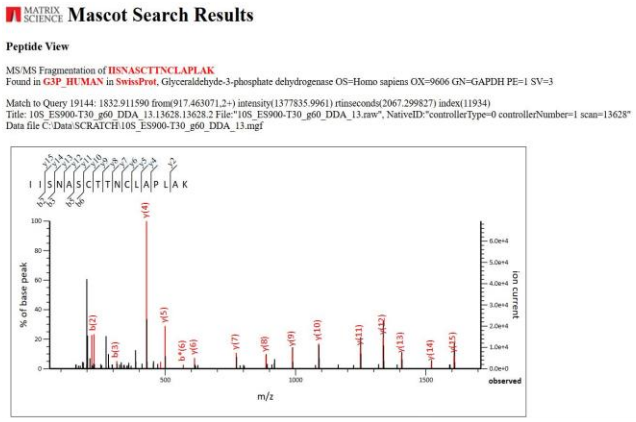

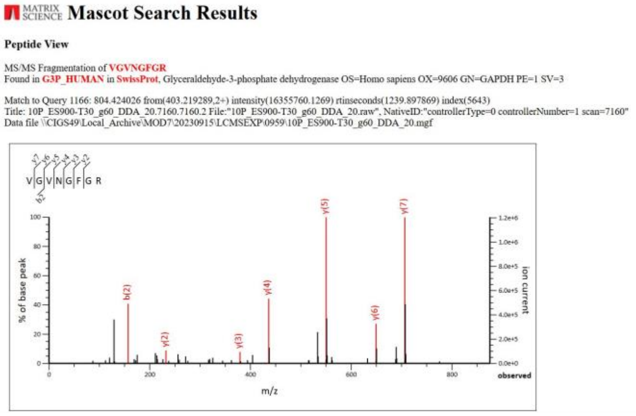

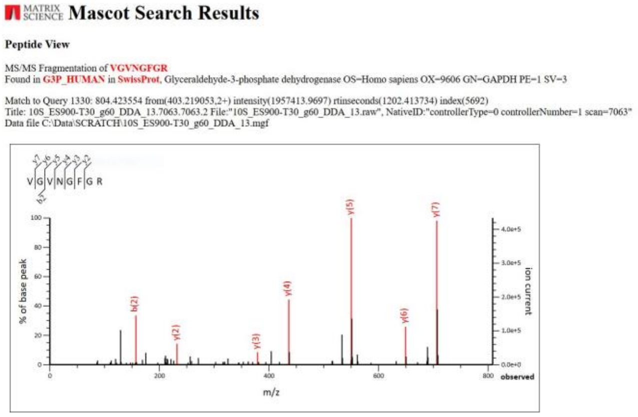

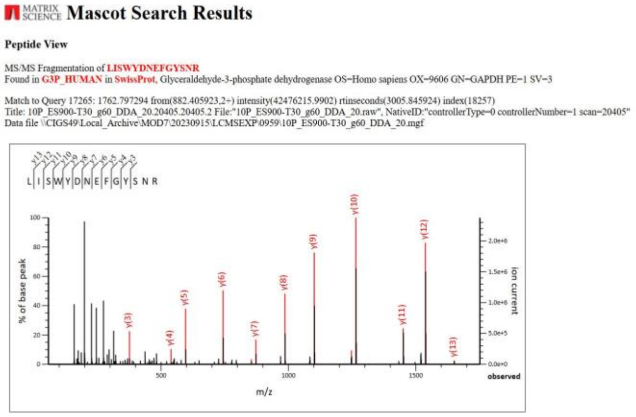

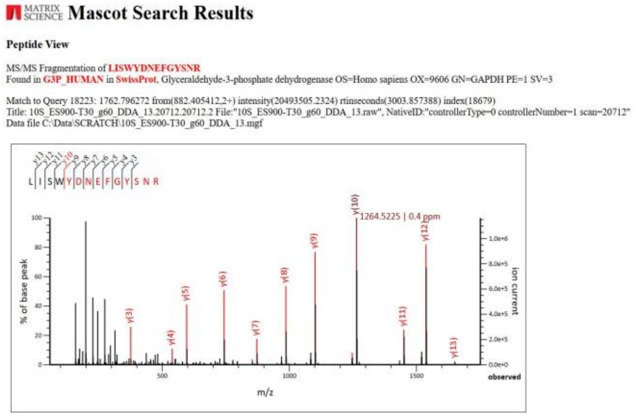

Supplement: Supplementary file 1 [file ijms-26-10441-s001.zip › Supplementary Figure S2.pdf]
